# ANGPTL3 possibly promotes cardiac angiogenesis through improving proangiogenic ability of endothelial progenitor cells after myocardial infarction

**DOI:** 10.1186/s12944-018-0835-0

**Published:** 2018-08-07

**Authors:** Fei Luo, Panyun Wu, Jingfei Chen, Yuan Guo, Jiangang Wang, Xiangping Li, Zhenfei Fang

**Affiliations:** 1 Department of Cardiovascular Medicine, The Second Xiangya Hospital, Central South University, 139 Middle Renmin Road, Changsha, 410011 Hunan China; 2 Department of Obstetrics and Gynecology, Xiangya Hospital, Central South University, Changsha, 410011 Hunan China; 3 Department of Cardiovascular Medicine, The Third Xiangya Hospital, Central South University, Changsha, 410011 Hunan China

**Keywords:** ANGPTL3, Angiogenesis, Endothelial progenitor cells, Myocardial infarction

## Abstract

Angiopoietin Like protein 3 (ANGPTL3) is at present considered as a central molecular target for therapy designed to reduce atherogenic lipids and atherosclerosis. However, concerns about the safety of inactivation of ANGPTL3 in patients with coronary artery disease (CAD) especially myocardial infarction (MI) have been raised. ANGPTL3 is reported to possess proangiogenic property. Angiogenesis is critical to the recovery of MI. Endothelial progenitor cells (EPCs) have multiple differentiation potential and play an important role in the angiogenesis post-MI. Promoting the function of EPCs could facilitate the angiogenesis and recovery of MI. Previous studies have shown that ANGPTL3 can promote angiogenesis in corneal of rats and promote angiogenesis of endothelial cells by binding to integrin α_ν_β_3_ receptors and promoting phosphorylation of protein kinase B (AKT). Our institution found that activated AKT can up-regulate the expression of microRNA-126 (miR-126), which can promote the proangiogenic ability of EPCs. The integrin α_ν_β_3_ receptors and AKT also express in EPCs and are closely related to proangiogenic function. Therefore, we hypothesized that ANGPTL3 could improve function of EPCs by binding to integrin α_ν_β_3_ receptors and up-regulating miR-126 expression via activating AKT, thus promoting the formation of new blood vessels, attenuating myocardial ischemia and improving heart function.

## Background

Coronary artery disease (CAD) is a leading cause of mortality worldwide, myocardial infarction (MI) is one of the most serious type of this disease. Remarkably good results have been achieved in the treatment of CAD by lipid-lowering strategy. Recent research reported individuals with Angiopoietin Like protein 3 (ANGPTL3) loss-of-function mutations demonstrate a 17% reduction in circulating triglycerides, a 12% reduction in low-density lipoprotein cholesterol, and a 34% reduction in odds of CAD, in contrast, high level of ANGPTL3 is positively associated with MI [1]. Inhibition of ANGPTL3 in dyslipidemic mice results in a dramatically decrease in atherogenic lipids, atherosclerotic lesion area and necrotic content [2]. In view of emerging results of human genetic analysis and preclinical studies, ANGPTL3 is at present considered as a central molecular target for therapy designed to reduce atherogenic lipids [3]. A variety of strategies have been used to genetically or pharmacologically inactivate ANGPTL3. In recent preclinical and clinical studies, CRISPR-Cas9 technology, antisense oligonucleotides and monoclonal antibody have been used to decrease circulating ANGPTL3 concentrations, resulting in an markable decrease in triglyceride-rich lipoprotein as well as low density lipoprotein cholesterol [4–6]. Therefore, ANGPTL3 seems to be a promising target for CAD treatment.

However, concerns about the safety of inactivation of ANGPTL3 in patients with CAD especially MI have been presented [7, 8]. Angiopoietin-like proteins show structural similarity to members of angiopoietin family proteins, which are closely related to angiogenesis. ANGPTL3, as one member of the ANGPTL family, is reported to promote angiogenesis and induce blood vessel formation [9]. Angiogenesis, the formation of new capillaries from pre-existing vessels, in the ischemic area after MI promotes cardiomyocyte survival and is believed to be essential to the recovery of patients with MI [10, 11]. Bonauer et al. [12] reported that inhibition of microRNA-92a enhances recovery of left ventricular function via accelerating blood vessel growth in a mouse model of acute MI. Miyahara et al. [13] reported transplantation of monolayered mesenchymal stem cells induces angiogenesis and improves cardiac function in rats with MI. These studies suggest a critical important role of angiogenesis for cardiac recovery after MI. However, there is little research about the function of ANGPTL3 in cardiac angiogenesis after MI. Previous study reported ANGPTL3 can promote angiogenesis in corneal of rats [9], suggesting that it may also promote cardiac angiogenesis after MI. If ANGPTL3 plays pivotal role in cardiac angiogenesis after MI, the high level of ANGPTL3 in MI patients may be beneficial for cardiac recovery and anti-ANGPTL3 therapy will be disadvantage [7, 8]. Therefore, we must be cautious with the use of inhibitors of ANGPTL3 and it is also important to illustrate the relationship between ANGPTL3 and cardiac angiogenesis after MI. In this hypothesis, we provide a possible proangiogenic mechanism of ANGPTL3 post-MI.

## Presentation of the hypothesis

Angiogenesis in response to ischemia after MI is critical important and believed to be initiated by endothelial progenitor cells (EPCs). The angiogenesis ability of EPCs mainly depends on its proliferation, adhesion, migration, and in vitro angiogenesis capacity. Previous study reported ANGPTL3 binds to integrin α_ν_β_3_ receptor and induces haptotactic endothelial cell adhesion and migration [9]. The angiogenesis associated receptor integrin α_ν_β_3_ is a kind of integrin receptor and expressed in cardiovascular endothelial cells. Integrin α_ν_β_3_ also was reported to express in EPCs and contribute to its migration [14]. Therefore, ANGPTL3 may also bind to the integrin α_ν_β_3_ in EPCs and facilitate EPCs to adhere and migrate.

Besides, ANGPTL3 could stimulate phosphorylation of AKT in human microvascular vein endothelial cells which contributes to its proangiogenic ability [9]. Our institution found that activated protein kinase B (AKT) signaling pathway increases the expression of microRNA-126 (miR-126) in EPCs and thereby improving angiogenesis [15]. It was reported transplantation of mesenchymal stem cells overexpressing miR-126 improves angiogenesis and cardiac function in MI mice [16, 17]. Consequently, we believe that ANGPTL3 can induce miR-126 expression by stimulating the AKT pathway.

Taken together, we hypothesize ANGPTL3 improves proangiogenic ability of EPCs via binding to integrin α_ν_β_3_ and increasing expression of miR-126 with an aim of providing more evidence for exploring the proangiogenic function of ANGPTL3 and insight into novel therapeutic target.

## Testing the hypothesis

We will design some experiments to test this hypothesis. ① C57BL/6 mice will be randomly treated with placebo or Angptl3 recombinant protein or monoclonal antibody, evinacumab, against Angptl3. After 24 h’ treatment, they will be conducted with MI surgery by ligating the left anterior descending coronary artery. After that, mice will continue the previous treatment for several days. At the end of the experiment, we will evaluate whether the recombinant protein can improve the cardiac function, infarct area and capillary density and illuminate the safety of evinacumab treatment in MI mice. Capillary density was determined by immunohistochemical staining with CD31 antibody as previously described [15]. We will also detect expression of integrin α_ν_β_3_ receptors, phosphorylated AKT, and miR-126. ② We will treat bone marrow-derived EPCs obtained from MI mice with or without ANGPTL3 recombinant protein, then detect the migratory ability, proliferative ability and in vitro angiogenesis of EPCs and also measure the expression of integrin α_ν_β_3_ receptors, phosphorylated AKT and miR-126.

## Abbreviations

AKT: Protein kinase B
ANGPTL3: Angiopoietin Like protein 3
CAD: Coronary artery disease
EPCs: Endothelial progenitor cells
MI: Myocardial infarction
miR-126: MicroRNA-126
